# Association between the severity of histopathological lesions and *Mycobacterium avium* subspecies *paratuberculosis* (MAP) molecular diversity in cattle in southern Chile

**DOI:** 10.3389/fvets.2022.962241

**Published:** 2023-01-12

**Authors:** Cristobal Verdugo, Diego Marquez, Enrique Paredes, Manuel Moroni, María José Navarrete-Talloni, Camilo Tomckowiack, Miguel Salgado

**Affiliations:** ^1^Instituto de Medicina Preventiva Veterinaria, Facultad de Ciencias Veterinarias, Universidad Austral de Chile, Valdivia, Chile; ^2^Center for the Surveillance and Evolution of Infectious Diseases (CSEID), Universidad Austral de Chile, Valdivia, Chile; ^3^Escuela de Graduados, Facultad de Ciencias Veterinarias, Universidad Austral de Chile, Valdivia, Chile; ^4^Instituto de Patología Animal, Facultad de Ciencias Veterinarias, Universidad Austral de Chile, Valdivia, Chile

**Keywords:** *Mycobacterium avium* subspecies *paratuberculosis*, MIRU-VNTR, histopathological lesions, score, dairy cattle

## Abstract

The objective was to evaluate the association between the severity of histopathological lesions caused by *Mycobacterium avium* subspecies *paratuberculosis* (MAP) infection and the molecular diversity of this pathogen. Blood, ileum, and mesenteric lymph node samples were collected at slaughter, from 1,352 adult cattle [source population 1 (SP1)]. In addition, 42 dairy herds (*n* = 4,963 cows) were followed for 2 years, and samples from compatible *paratuberculosis* clinical cases [source population 2 (SP2)] were collected. MAP infection was confirmed using an ELISA test, liquid media culture, and PCR. Isolates were genotyped using five MIRU-VNTR markers. Tissues from confirmed samples were subjected to a histopathological examination. A histopathological severity score (HSS) system was developed and used to grade (0 to 5) the magnitude of lesions caused by MAP. In general, the HSS system assesses the number of foci and degree of macrophage infiltration, together with the presence of multinucleated giant cells (MGCs) and acid-fast bacilli (AFB), in addition to the fusion of the intestinal villi and hyperplasia of the crypts. Despite the large sampling effort, only 79 MAP isolates were successfully genotyped, where 19 different haplotypes were described. A mixed-effect Poisson regression model was used to assess the relationship between haplotypes and HSS values. The model was controlled by animal age, and the farm was used as a random effect. Haplotypes were grouped based on their relative frequency: the most frequent haplotype (group *i*, 49.4%), the second most frequent haplotype (group *ii*, 12.7%), and all other haplotypes (group *iii*, 37.9%). Model outputs indicated that group *i* had significantly higher HSS values than group *iii*. In addition, group *i* was also associated with higher optical density (OD) values of the ELISA test. These results support the existence of differences in pathogenicity between MAP haplotypes. However, results were based on a relatively small sample size; thus, these should be taken with caution. Despite this, study findings suggest that haplotypes would be associated with differences in disease progression, where the dominant haplotype tends to generate more severe lesions, which could be linked to a greater shed of MAP cells than non-dominant haplotypes, increasing their chances of transmission.

## 1. Introduction

*Paratuberculosis* (Ptb) or Johne's disease is a chronic granulomatous enteritis caused by *Mycobacterium avium* subsp. *paratuberculosis* (MAP), which affects all ruminant species in practically all countries around the world (1, 2). Among domestic ruminants, MAP infection is widespread, where high herd-level prevalence has been reported in dairy cattle herds in the USA, Denmark, Ireland, Chile, and Italy, for example (3–7). From an economic point of view, MAP infection has been associated with important losses, mainly due to a decrease in milk production and the early elimination of animals (8). For example, in the USA dairy sector, an annual loss of US$ 200 to US$ 250 million was estimated in the early 2000s (9).

Current knowledge indicates that MAP is transmitted between animals mainly by the fecal–oral route (10), where the primary source is infected adult animals. In this sense, the infection spreads by the elimination of MAP in the feces of infected animals (11), which can contaminate soil, grass, food, and water (12). In addition, milk from infected females represents another source of infection. Moreover, pseudo-vertical transmission has also been described, where a calf is infected during delivery, or soon after (13). Delivery together with the 1^st^ weeks of life represents the main infection risk period for susceptible animals (14). Despite MAP transmission to a new host commonly occurring during the 1^st^ months of life, clinical disease is commonly observed in animals between 3 and 5 years, usually in the postpartum period. Although the clinical phase can lead to animal death or culling, a significant proportion of infected animals remains in a subclinical state throughout their productive life (11). Currently, there is not a complete understanding of why some animals will progress toward a clinical phase, while others will not show any sign of the disease. It is assumed that the progression toward the clinical phase is related to host and environmental factors, as well as factors related to the agent (15). Among the factors associated with the host, variation in the presentation of the clinical phase has been explained by the age at which the first infection occurs, the infective dose, the stress caused by associated diseases, births, and/or lack of adequate feed, in addition to host genetic resistance, among other causes (11, 16, 17). In contrast, there is little information on the effect of the agent on the presentation of the clinical disease, and this is mainly based on laboratory-based studies. In particular, it has been observed that MAP survival in macrophages in cattle would be somewhat determined by the type of strain (18), which also could determine variations in the host's immune response (19, 20). Similarly, studies in deer and sheep have reported changes in virulence, according to the type of MAP strain present (21–23). In general, the study of factors affecting the presentation of clinical disease has been hampered by the difficulties of conducting longitudinal studies for this disease. Specifically, clinical disease is characterized by a long incubation period and a relatively low incidence despite its widespread infection (7). Thus, the necessary number of animals to be enrolled and the duration of the study required to observe the effect of relevant factors are prohibitive. In contrast, MAP is characterized by a low genetic diversity, where it is commonly considered that classical molecular techniques lack enough discriminatory power to conduct meaningful epidemiological studies. Despite this, two of the most employed typing methods, short sequence repeat (SSR) and the mycobacterial interspersed repetitive unit variable-number tandem repeat (MIRU-VNTR), have been used to describe MAP diversity, host preferences, and the association with histopathological lesions (24–27). In particular, those methods allow the typing of MAP isolates based on the number of copies of specific markers in their genome (28, 29).

Although clinical signs are commonly not observed, histopathology techniques allow to study the variations in the lesions caused by MAP in different tissues, and at different stages of the infection. Specifically, it is possible to classify and rate the histopathological changes according to the severity of the lesions, mainly based on the number of macrophages, type of inflammatory cells, and level of intestinal villi damage (30). Thus, a histopathology severity score (HSS) system for the graduation of lesions was proposed by Clarke et al. (31, 32). That system has been used for the study of MAP infection in red deer (*Cervus elaphus*), with the aim of classifying and comparing histopathological differences between infected subjects (23). The combination of MAP molecular diversity and histopathological data could provide relevant information on the association between MAP subtypes and the evolution of the infection toward the clinical stage, where this approach could elucidate, in part, if some MAP subtypes/strains are more pathogenic than others. In this way, the objective of this study was to evaluate the association between the severity of histopathological lesions in cattle and the MAP subtype isolated from those lesions.

## 2. Materials and methods

### 2.1. Target population and experimental design

The target population was defined as adult dairy cattle (8 teeth) from farms in southern Chile (Los Ríos and Los Lagos regions), which concentrate approximately 78% of Chile's milk production (33). Two source populations (SPs) were considered. The first one (SP1) corresponded to animals that met the mentioned age and origin criteria, and that were delivered to the main meat-processing plant in Los Ríos Region over the sampling period. The second population (SP2) corresponded to field-level euthanized cattle that presented clinical signs compatible with Ptb (from dairy farms located in the study regions). From both source populations, biological samples corresponding to ligated segments of the ileum terminal portion were collected along with its associated lymph node, in addition to blood obtained during the animal bleeding (SP1), or by puncture of the coccygeal vein (SP2).

In the case of SP1, all cows that met the inclusion criteria were subjected to sample collection. Specifically, ileum and mesenteric lymph node (MLN) samples were collected in individual nylon bags, after handling with sterilized materials. Once identified and labeled, together with the associated blood samples, they were transported in cooler containers (within the next 6 h) to the Laboratory of Infectious Diseases of the Preventive Veterinary Medicine Department, at the Austral University of Chile. Upon arrival, blood samples were subjected to ELISA testing, and tissue samples from ELISA-positive animals were eligible for further histopathological and microbiology analyses. Specifically, MLN and ileum samples were divided into two portions. One portion was placed in a jar with 10% formalin for histopathological examination at the Animal Pathology Department of the Austral University of Chile, while the remaining portion was subjected to individual microbiological culture in a liquid medium. Positive culture samples were subjected to a PCR assay to confirm the presence of MAP. Finally, isolates from confirmed samples were typed using the MIRU-VNTR method. In contrast, tissues associated with negative ELISA samples were discarded following the biological waste disposal protocol of the Austral University of Chile. The use of biological material from abattoirs, as well as the sampling and euthanasia of clinical animals at the farm level, were authorized by the Animal Bioethics Committee of the Austral University of Chile (N°194/2014).

### 2.2. Serology, microbiology, and molecular typing analyzes

All blood samples were analyzed using the ELISA IDEXX *Paratuberculosis* Screening Ab Test. Samples were tested according to the manufacturer's instructions for cattle sera. Results were interpreted by measuring the optical density (OD) using an ELISA reader (BioTek Instrument, Inc. VT., USA) with a 450 nm filter. The absence/presence of anti-MAP antibodies was determined by calculating the OD sample to the positive ratio (S/P) with a cut-off of 0.50. Tissue microbiological culture was performed on samples from ELISA-positive animals. The culture was carried out using the MGIT Bactec system (Becton Dickinson, Sparks, MD), where tissues were prepared following the manufacturer's recommendations and inoculated in tubes with MGIT ParaTB media containing supplements and antibiotics according to the manufacturer's instructions (Becton Dickinson, Sparks, MD). Specifically, each tube contained 7 ml of Middlebrook 7H9 broth base modified with Mycobactin J and a fluorescent oxygen indicator embedded in silicone at the bottom of the tube. For each tube, 800 μl of MGIT ParaTB supplement, 500 μl of egg yolk suspension (Becton Dickinson, Sparks, MD), and 100 μl of VAN cocktail (Becton Dickinson, Sparks, MD) were added, resulting in final concentrations of 10 μg / ml vancomycin, 40 μg/ml of amphotericin B, and 60 μg/ml of nalidixic acid. Each inoculated MGIT tube was introduced into the MGIT Bactec system and incubated at 37°C for 49 days or until the instrument yielded a positive signal. In that case, the presence of MAP was confirmed through a PCR assay targeting the IS900 insertion sequence following the protocol described by Salgado et al. (34). Finally, in samples where the presence of MAP was confirmed, the bacterium was isolated and subjected to molecular typing using the MIRU-VNTR method (35). In particular, five markers were targeted (**VNTR**-292, **VNTR**-X3, **VNTR**-47, **VNTR**-3, and **VNTR**-25), which in a previous study have shown to be the markers with the greatest discriminatory power, under the conditions present in Chile (36). For each marker, a conventional PCR assay was carried out following the protocol proposed by Thibault et al. (35), and the number of repetitions was estimated through its molecular weight by electrophoresis. The unique combination of repetitions of each marker represents a specific haplotype profile, which was used as an indicator of a given MAP subtype.

### 2.3. Histopathology and severity score system

Tissue samples from ELISA and culture-positive animals were subjected to histopathological assessment. MLN and ileum tissues were fixed in formalin 10% and processed following the methodology described by Luna (37), where tissues were laminated obtaining sections 4–5 μm thick and treated with hematoxylin/eosin and Ziehl–Neelsen staining. Individual cassettes were prepared for ileum and MLN samples, thus handling separate plates for each tissue. These were examined using optical microscopy, under various magnifications (4X, 10X, 40X, 60X, and 100X). The objective of this analysis was to describe the lesions present in the selected tissues and to assign a weighted score that represents the magnitude of histopathological visible lesions. To perform this graduation, the HSS proposed by Clark et al. (31, 32) for cervids was used as a model and adapted to cattle in this study. In general, lesions were scored based on the number of foci and degree of macrophage infiltration, together with the presence of multinucleated giant cells (MGCs) and acid-fast bacilli (AFB), in addition to the fusion of the intestinal villi and hyperplasia of the crypts. This HSS system uses an ordinal scale from zero (no damage compatible with Ptb) to five. Table 1 shows the histopathological criteria used to assign each score to the lesions. Supporting images have been included as Supplementary material A.

**Table 1:** General criteria description of the histopathological severity score (HSS) system for lesions of ileum and mesenteric lymph nodes (MLN) of dairy cattle infected with *Mycobacterium avium* subsp. *paratuberculosis*.

**Grade**	**Ileum**	**MLN**
0	No lesions compatible with Ptb	No lesions compatible with Ptb
1	Focal to multifocal (1–5 foci) and mild (< 20% of the tissue section) infiltration of macrophages, with or without multinucleated giant cells (MGC) within the intestinal mucosa and/or Peyer's patches. Up to 10 acid-fast bacilli	Focal to multifocal (1–5 foci) and mild (< 20% of the tissue section) infiltration of macrophages, with or without multinucleated giant cells (MGC) within the tissue (except the capsule). Up to 10 acid-fast bacilli
2	Multifocal (>5 foci) and moderate (20–50% of the tissue section) infiltration of macrophages, with or without MGC within the intestinal mucosa and/or Peyer's patches. Up to 10 acid-fast bacilli per macrophage or MGC	Multifocal (>5 foci) and moderate (20–50% of the tissue section) infiltration of macrophages, with or without MGC within the tissue (except the capsule). Up to 10 acid-fast bacilli per macrophage or MGC
3	Focally-extensive (coalescing foci with poorly delineated margins) and moderate (20–50% of the tissue section) infiltration of macrophages, with or without MGC within the intestinal mucosa and/or Peyer's patches. Up to 10 acid-fast bacilli per macrophage or MGC	Focally-extensive (coalescing foci with poorly delineated margins) and moderate (20–50% of the tissue section) infiltration of macrophages, with or without MGC within the tissue (except the capsule). Up to 10 acid-fast bacilli per macrophage or MGC
4	Diffuse (ill-defined and poorly delineated) and severe (>50% of the tissue section) infiltration of macrophages, with high numbers of MGC within the intestinal mucosa and/or Peyer's patches. More than 10 acid-fast bacilli per macrophage or MGC. Marked atrophy and fusion of intestinal villi and crypt hyperplasia may be present	Diffuse (ill-defined and poorly delineated) and severe (>50% of the tissue section) infiltration of macrophages, with high numbers of MGC within the tissue (except the capsule). More than 10 acid-fast bacilli per macrophage or MGC
5	Organized granulomas with a necrotic center within the intestinal mucosa and/or Peyer's patches. More than 10 acid-fast bacilli per macrophage or MGC. Marked atrophy and fusion of intestinal villi and crypt hyperplasia may be present	Organized granulomas with a necrotic center within the tissue (except the capsule). More than 10% acid-fast bacilli per macrophage or MGC

### 2.4. Data analysis

Haplotype frequencies were cross-tabulated by source population, along with descriptive statistics of HSS values for each SP and sample type. The weighted Cohen's kappa value was calculated to estimate the agreement between HSS values, from pairs of ileum and MLN samples. The Mann–Whitney *U*-test was used for the comparative analysis of the HSS values between ileum and MLN samples, or between SP. The Simpson's diversity index (DI) was calculated to assess the discriminatory power of the MIRU-VNTR markers used (all isolates were considered, independently of the farm of origin). A minimum spanning tree (MST), together with the proportional similarity index (PSI), was used to evaluate differences in haplotype frequency distributions between the SP. The association between MAP haplotypes and the severity of compatible lesions was performed using a mixed-effect Poisson regression model, where the HSS value corresponded to the response variable, whereas the haplotype profile corresponded to the explanatory variable. For this purpose, the model was controlled by animal age, and the haplotype profiles were grouped into three categories (groups), being: (*i*) the most frequent haplotype, (*ii*) the second most frequent haplotype, and (*iii*) all other haplotypes. Given that multiple samples could be collected from the same dairy, farm-ID was used as a random effect to control for collinearity between samples. In addition, the association between the haplotypes groups (*i, ii, and iii*) and the time to detection (TTD) in the MGIT Bactec system, or to the S/P value of the ELISA test were assessed using a Kruskal–Wallis test. *Post-hoc* comparisons were performed using a pairwise Wilcox test. All statistical analyses were carried out using R software v 4.0.2 (38).

## 3. Results

In SP1, 1,352 cows were sampled at the abattoir level, from 44 different farms, between July and August 2017. From those sampled animals, 106 were ELISA positive, while the presence of MAP was confirmed in 74 animals. In contrast, SP2 samples were obtained from a longitudinal MAP study (7), which follow-up 42 dairy farms with a total population of 4,963 adult cows. Although a 1.1% annual incidence of clinical disease was estimated in this population (7), tissue samples for histopathology were obtained from only 11 confirmed clinical cases (from 11 different farms), mainly due to follow-up difficulties that precluded timing sample collection before animals were culled by farmers. In this way, both subpopulations yielded a total of 85 MAP-confirmed samples, with tissue samples available, from which successful MIRU-VNTR typing was obtained from a total of 79 samples.

**Figure 1:**
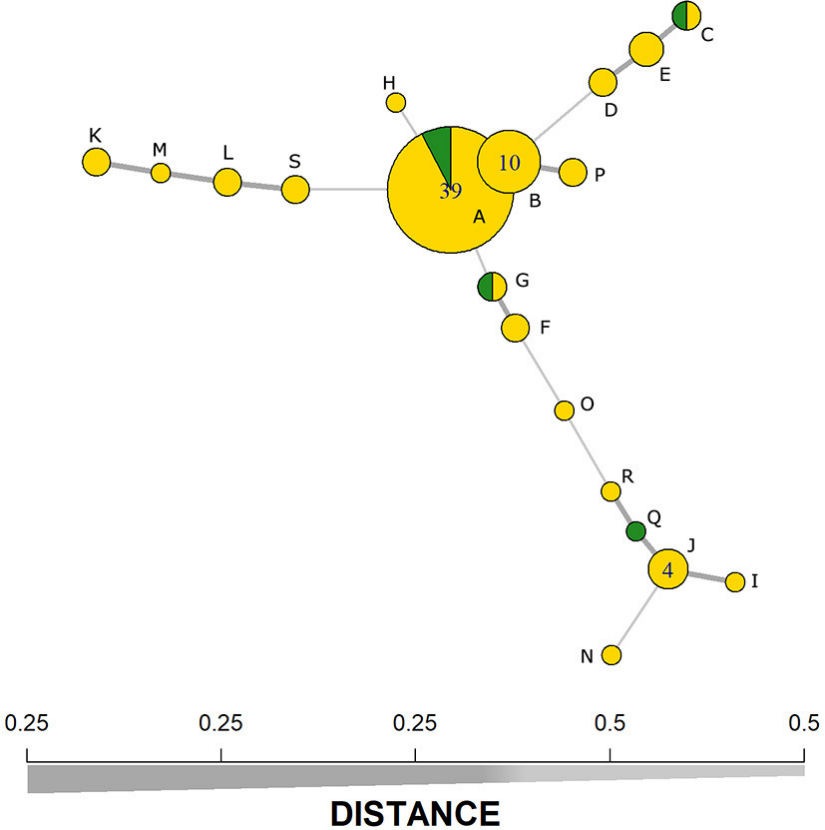
Minimum-spanning tree based on five MIRU-VNTR markers representing the clustering of 79 *Mycobacterium avium* subspecies *paratuberculosis* (MAP) isolates from subpopulation 1 (yellow) and subpopulation 2 (green). Line thickness represents the genetic distance between the haplotypes (x-axis). Alphabetic letters represent the haplotypes as presented in Table 2.

The typing method divided MAP isolates into 19 profiles (Table 2), with a DI of 0.74 for the five MIRU-VNTR markers used. Despite this, the two most dominant haplotypes (groups *i* and *ii)* represented 49 and 13% of the isolates, respectively. The SP1 presented 18 different haplotypes (haplotype Q was not observed), whereas SP2 had four different haplotypes (A, E, F, and Q). Moreover, the MST analysis indicates that the two main haplotypes present a very low genetic distance between them (Figure 1), where only one repetition in the VNTR-292 marker differentiates them. The comparison of haplotype distributions between subpopulations (SP1 vs. SP 2) indicated a probability (PSI) of 0.21 (95% CI: 0.03–0.45) of both subpopulations sharing the same haplotypes.

**Table 2:** Copy numbers for the five MIRU-VNTR markers used for the strain-typing of *Mycobacterium avium* subspecies *paratuberculosis* (MAP) isolates.

**Haplotype**	**VNTR 292**	**VNTR X3**	**VNTR 25**	**VNTR 47**	**VNTR 3**	**Frequency**	**%**	**Group**
A	3	2	3	3	2	39	49.4	*i*
B	4	2	3	3	2	10	12.7	*ii*
C	1	1	3	3	2	2	2.5	*iii*
D	2	1	3	3	2	2	2.5	*iii*
E	3	1	3	3	2	3	3.8	*iii*
F	3	2	2	3	1	2	2.5	*iii*
G	3	2	2	3	2	2	2.5	*iii*
H	3	2	3	4	2	1	1.3	*iii*
I	3	2	4	3	1	1	1.3	*iii*
J	3	2	4	3	2	4	5.1	*iii*
K	3	3	3	3	1	2	2.5	*iii*
L	3	3	3	3	2	2	2.5	*iii*
M	3	3	3	3	3	1	1.3	*iii*
N	3	4	4	3	2	1	1.3	*iii*
O	4	2	2	3	3	1	1.3	*iii*
P	4	2	3	3	1	2	2.5	*iii*
Q	4	2	4	3	2	1	1.3	*iii*
R	4	2	4	3	3	1	1.3	*iii*
S	4	3	3	3	2	2	2.5	*iii*

Absolute and relative frequency of the 19 haplotypes formed by the combination of the five MIRU-VNTR markers. And grouping of haplotypes. Group *i*: Most common haplotype, Group *ii*: second most frequent haplotype, Group *iii*: rest of the observed haplotypes combined into a single group.

**Figure 2:**
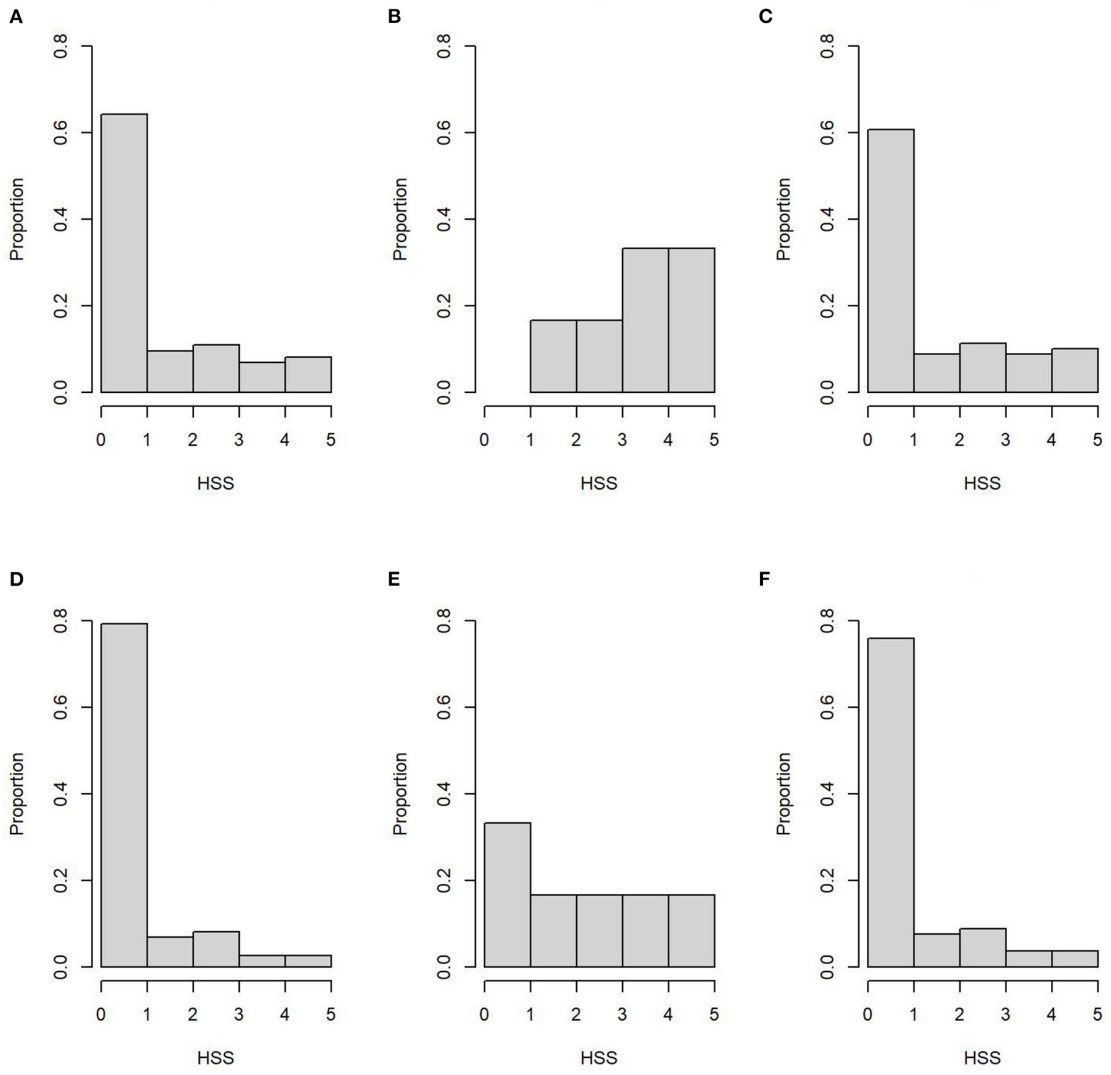
Histopathology severity score (HSS) distribution of ileum samples (superior row) for the subpopulation 1 **(A)**, subpopulation 2 **(B)**, and both subpopulations **(C)**. Bottom row represents HSS distribution of mesenteric lymph nodes samples from subpopulation 1 **(D)**, subpopulation 2 **(E)**, and both subpopulations **(F)**.

The HSS distributions across sample type and SP are shown in Figure 2. Moreover, age and HSS descriptive statistics are shown in Table 3, where no significant age differences (*p* = 0.84) were observed between SP. Conversely, HSS values presented a statistical difference between SP, for both sample types, where SP2 presented significantly higher scores than SP1 (Table 3). Similarly, HSS values between sample types showed a significant difference (*p* < 0.001), where intestine samples presented higher HSS values than MLN samples. In particular, HSS values of ileum and MLN samples had a weighted kappa value of 0.58 (95% CI: 0.53–0.62), indicating weak agreement between them, despite having been obtained from the same subject. Additional information on HSS and OD distributions by haplotype is presented in Supplementary material B. The multivariate analysis, which modeled HSS values across the grouped haplotypes, indicated a statistical difference between the most frequent haplotype (group *i*) vs. all other haplotypes (group *iii*), where the dominant haplotype presented significantly higher HSS values (Table 4). Finally, no statistical differences were observed between groups (*i, ii*, and *iii*) and TTD. However, significantly higher OD values were observed in group *i* when it was compared with values from group *iii* (*p* = 0.049).

**Table 3:** Descriptive and analytical statistics of histopathology severity score (HSS) comparison across distribution across subpopulation origin, sample types and age.

**Variables**	**Min**	**1^st^Q**	**Median**	**3^rd^Q**	**Max**
**Overall**					
-Ileum^a^	0	0	1	3	5
-MLN^a^	0	0	1	1	5
**SP1**					
-Ileum^b^	0	0	1	3	5
-MLN^c^	0	0	1	1	5
**SP2**					
-Ileum^b^	0	3.3	4	4.8	5
-MLN^c^	0	1.3	2.5	3.8	5
**Age**					
-SP1	2	5	6	7	12
-SP2	4	5	5	5.8	9
All	2	5	5	7	12

MLN, Mesenteric lymph node; SP1, sub-population 1; SP2, sub-population 2.

a Wilcoxon signed rank test with continuity correction, V = 599.5, *p*-value = 0.0007366.

b Wilcoxon rank sum test with continuity correction, W = 74.5, *p*-value = 0.005959.

c Wilcoxon rank sum test with continuity correction, W = 111.5, *p*-value = 0.03576.

**Table 4:** Mixed effects Poisson regression model results including predictor estimates, standard deviation (sd), statistic (z-value), and *p*-value.

**Predictor**	**Estimate**	**sd**	**z-value**	***p*-value**
Intercept	0.84	0.32	2.63	0.01
**Group**				
-*i*	Ref			
-*ii*	−0.01	0.32	−0.02	0.98
-*iii*	−0.42	0.20	−2.14	0.03
Age	−0.04	0.05	−0.90	0.37

The histopathology severity score (HSS) corresponded to the response variable, whereas farm-ID was used as a random effect. Group *i*, Most common haplotype; Group *ii*, second most frequent haplotype; Group *iii*, rest of the observed haplotypes combined into a single group.

## 4. Discussion

This research aimed to assess the relationship between MAP haplotypes and the severity of histopathological lesions associated with this infection. The novelty of the present research lies in the combination of a well-established genotyping technique such as MIRU-VNTR, with an HSS system. This allowed a semi-quantitative assessment of the severity of the lesions in relation to the disease progression. Model outcomes indicate that the dominant haplotype was associated with the presentation of more severe histopathological lesions and elicited a stronger immune response in comparison to the grouped non-dominant haplotypes (group *iii*). Thus, results support the hypothesis that there are pathogenic differences between MAP haplotypes. However, the results were based on a relatively small sample size; thus, larger sampling efforts would be required to confirm these findings. In addition, the HSS system would require validation by other researchers, to measure potential interpretation differences between research groups that could lead to differences in HSS values. Therefore, further research on this subject is needed. Despite these limitations, the study's findings suggest that haplotypes are associated with differences in disease progression. In contrast, it is important to highlight that group *iii* is composed of 17 haplotypes (out of 19), with individual frequencies between 1.3 and 5.1%, representing 37.9% of all observed haplotypes. Thus, it can be inferred that MAP isolates belonging to group *iii* have a lower transmissibility than isolates from the two dominant haplotypes. Moreover, it should be noted that evidence supporting differences in the ability to cause disease between MAP isolates has not been robustly documented and it has been mainly based on *in vitro* experiments using animal and cellular models (18–23), where MAP isolates were typed through methods with relatively lower discriminatory power. Conversely, the present results are based on field study. Möbius et al. (25) conducted a relatively similar study, assessing the relation between genotyped field isolates and macroscopic lesions, reporting a statistical association between macroscopic lesions and individual combined genotypes. Specifically, they observed that one of the most frequent genotypes around the world [IS900-RFLP-(BstEII)-Type-C1] was more prevalent in animals with macroscopic lesions. In this sense, our approach goes further, increasing the level of detail in the assessment of the lesions associated with MAP infection.

Even though the study was based on the sampling of two different source populations, where more than 1,300 animals were sampled in SP1 and 42 dairy farms in SP2 (*n* = 4,963 cows) were followed for more than 2 years (7, 39), only 79 fully genotyped isolates were obtained. This highlights the difficulty of conducting molecular epidemiology studies for MAP infection, which require large sample sizes. The relatively limited number of isolates typed was the main reason for grouping less frequent haplotypes into a single category, to perform a proper statistical analysis. This grouping could inadvertently have diluted the effect of some non-dominant but highly pathogenic haplotypes, being a limitation of the statistical analysis performed. In contrast, the model did not detect statistical differences between groups *i* and *ii*, which could be explained by the low genetic distance between the two most dominant haplotypes.

The five VNTR markers separated MAP isolates into 19 different haplotypes with a DI of 0.74, indicating an acceptable level of discrimination to conduct epidemiological studies. The observed diversity is comparable to reports from previous studies using this typing method. For example, Thibault et al. (35), using eight MIRU-VNTR markers, classified 183 isolates (from 10 different countries) into 21 subtypes. Similarly, Stevenson et al. (26) subdivided 147 isolates (from seven countries) into 23 different haplotypes. Furthermore, Moebius et al. (40) reported 15 haplotypes from a collection of 71 isolates from Germany, using 10 markers. Likewise, van Hulzen et al. (41) using seven markers subtyped 52 isolates into 17 haplotypes in the Netherlands. Whereas, Castellanos et al. (42) reported 12 haplotypes from a collection of 70 isolates in Spain. Likewise, Verdugo et al. (43) described 17 haplotypes in New Zealand, from 238 isolates retrieved from three different livestock species. In Chile, a previous non–peer-reviewed study by Verdugo et al. (36) identified the same 19 haplotypes described in the present study but using a collection of 122 isolates from different sources (wildlife, livestock, and environmental samples). Similarly, to the present research, previous studies have also reported the presence of one or two high-frequency (or dominant) haplotypes (26, 27, 40–42), indicating that those dominant haplotypes present a better fitness than the rest of the haplotypes described in their respective population. In this sense, the present study showed that the dominant haplotype tends to be associated with higher HSS and OD values, which would indicate that isolates from this group are capable of speeding up the infection progression. Since TTD was non-significantly different between haplotypes, this phenomenon could be associated with a more extended bacterial invasion of target tissues, shedding larger bacteria loads into the environment, and thus out competing the other reported haplotypes. However, the possibility that the liquid medium does not provide the same conditions as at the cellular level cannot be ruled out, thus preventing the expression of differences in replication rates if they exist. The early cellular and molecular processes that determine whether the infection will be eliminated or persist have not been fully understood. Nevertheless, it is known that a high cellular immune response would be characteristic of initial disease states, and at the same time responsible for maintaining a subclinical state. The persistence of a low number of intracellular MAP cells over long periods may reflect a balance between host defenses and pathogen resistance, producing subclinical cases. Conversely, a strong humoral response is associated with more advanced stages of infection (44), where MAP organisms present in the intestinal epithelium and MLN are in an exponential growth phase, characterized by fast cellular replication, leading to heavy bacterial shedding, with loads of 10^3^ to 10^4^ MAP CFU per gram of feces (45).

The HSS comparison between source populations or sample types also presented statistical differences. In particular, SP2 had significantly higher HSS than SP1, which could be expected given that SP2 samples were obtained from animals with clinical signs of Ptb. It is important to highlight that tissue samples were blinded during the histopathology assessment, indicating that the proposed HSS system was able to differentiate between disease stages. In contrast, ileum and MLN also presented significant differences and poor agreement between them, despite having been obtained from the same animal, where ileum samples presented higher HSS than MLN samples. This difference could be explained by the MAP infection route, where after being ingested orally, MAP invades the host at the level of the terminal ileum, through Peyer's patches, where they are phagocytized by macrophages, in which this pathogen has the ability to survive and reproduce (46). Therefore, differences in HSS between sample types possibly represent the longer time that MAP has been present in the ileum in comparison to the MLN.

Traditionally, lesions have generally been classified as mild, moderate, and severe, where this classification is commonly based on the number of macrophages, the number and spread of MGC, the type of inflammatory cells, and the degree of involvement of intestinal villi (30). Injury severity graduation systems have been developed to have more objective protocols for the classification of histopathological lesions and they have been implemented mainly on red deer (*Cervus elaphus*) (23, 47, 48). For example, Clark et al. (31, 32) proposed a system based on an increasing scale of quantitative weighting based on the presence and number of AFB, macrophages, and MGC, as well as the presence and dispersion of granulomas and the level of mineralization of the lesions. For the present study, a classification system was developed to weigh the magnitude of histopathological lesions, considering the main findings described in published studies (23, 30–32, 47–50). This HSS integrated elements described for Ptb in cervids, but they were adapted to fit the characteristic histopathology evolution of MAP infection in cattle. In this regard, it is important to note that deer present a rapid and acute course of the disease, where clinical cases are commonly observed at an early age (< 12 months) (51). This may mean an accelerated progression of histopathological lesions, with the consequent presentation of a greater number of different stages. Therefore, the faster disease progression determines a larger number of stages that can be defined for the score-classification of histopathological lesions in this species. In this line, Clark et al. (31) originally used 13 categories, which were later reduced to seven plus three intermediate categories (32). Conversely, in cattle, a slower disease progression has been described, allowing to define more delimited stages; thus a relatively smaller number of categories can be used to rank Ptb-related lesions. In this regard, Clark et al. (32) considered intermediate categories (“+0.5”) for scores 4 to 6, when lesions were observed in the villi. In contrast, the present HSS villi lesion was reserved for scores 4 or 5. In addition, Clark et al. (32) considered an increase in the graduation for scores 2 and 3, when there was evidence of lesions in several segments of the intestine. Conversely, the present HSS did not consider such an increase because only one segment of the ileum was collected. This protocol was chosen because we only had 60 seconds to collect the tissue samples due to restrictions imposed by the meat plant, in order to maintain their processing speed. Finally, it is important to highlight that an excessive number of categories could reduce the study power, requiring an even larger sample size to be able to find statistical associations. In this way, the HSS used in this research provides a balance between the capacity of the system to differentiate different stages of the infection at the tissue level and the ability to conduct proper statistical comparisons.

*Paratuberculosis* is a chronic disease, where the age of infection determines how long MAP has been able to progress and cause damage. Thus, variations in HSS could be explained by age heterogenicity rather than haplotype differences, potentially biasing study results. However, the regression analysis was controlled by animal age; hence, model outputs considered the age effect on the HSS distribution. In addition, current knowledge indicates that the major risk of infection occurs in the 1^st^ months of life (15, 52). Therefore, it could be assumed with a certain degree of confidence, a large proportion of the sampled animals got infected at a relatively closer age. Given those two elements, we considered that the bias effect of age differences between animals was negligible. In this same line, another potential bias could be a product of the sampling design, where MAP isolates were obtained from ELISA-positive animals. However, in sub-clinically infected animals, the sensitivity of the ELISA test is relatively low (53, 54). Thus, during the sampling process, a proportion of the infected population was missed. Given that study results have shown that less frequent haplotypes tend to be associated with lower S/P values, it is fair to assume that an important proportion of those false negative animals would have been infected with some of the non-dominant haplotypes. This could reduce the observed haplotype diversity, where haplotypes that tend to elicit a low immune response would have been not detected during the screening stage. Despite this, we consider that the effect of those missed haplotypes is limited in the overall results of the study, given that low-frequent haplotypes were lumped into a single group for data analysis.

The present study reported the association between the MAP dominant haplotype and the presence of more severe histopathological lesions, supporting the hypothesis of pathogenicity differences between MAP isolates. However, the clinical manifestation of MAP is multifactorial, where host genetic, environmental, and microbial elements interact leading to different manifestations of this disease. Thus, the present finding probably represents one component in a web of interrelated factors determining disease progression. Nevertheless, this research provides novel evidence on the pathogenic differences between MAP isolates, contributing to a better understanding of Ptb epidemiology.

## Data availability statement

The raw data supporting the conclusions of this article will be made available by the authors, without undue reservation.

## Ethics statement

The animal study was reviewed and approved by Animal Bioethics Committee of the Austral University of Chile (N°194/2014). Written informed consent was obtained from the owners for the participation of their animals in this study.

## Author contributions

CV was contributed in study design, manuscript writing, and data analysis. DM was contributed in sample collection, histopathological assessment of samples, and writing. EP, MM, and MN-T were in charge of developing and adjusting the HSS system. CT was in charge of genotyping and molecular analysis. MS was in charge of overseeing all laboratory analyses. All authors contributed to the article and approved the submitted version.
