# Supplementary material for: Association between the severity of histopathological lesions and Mycobacterium avium subspecies paratuberculosis (MAP) molecular diversity in cattle in southern Chile
Source: Front Vet Sci. 2023 Jan 12;9:962241. doi: 10.3389/fvets.2022.962241 (PMC9878319; doi:10.3389/fvets.2022.962241)
Supplement: Supplementary file 1 [file Data_Sheet_1.PDF]

Supplementary Material A

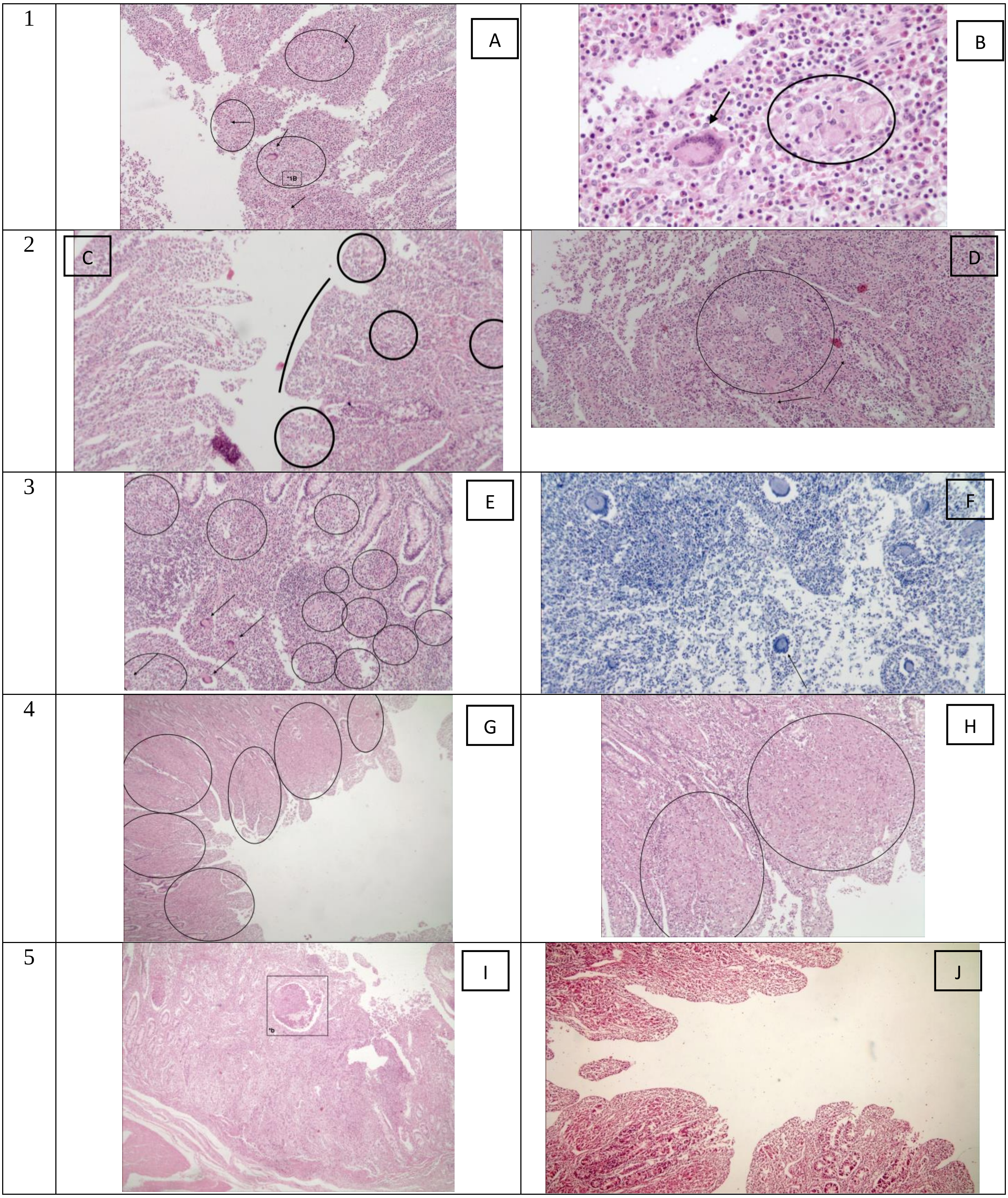

Figure A. Grade 1: Ileum sample, macrophage infiltrate is observed (mainly macrophages and lymphocytes, in addition to abundant eosinophils and some erythrocytes) on the lamina propria of the intestinal mucosa (circles) with some multinucleated giant cells (MGC) (arrows). Hematoxylin & eosin (HE) stain 10x.
Figure B (zoom of Figure A): Grade 1, macrophage infiltrate on the intestinal mucosa (circle), and MGC (arrow). HE stain 40x.
Figure C. Grade 2: Ileum sample with moderate multifocal macrophage infiltrate in the lamina propria of the intestinal mucosa (circles). A slight fusion of intestinal villi can also be observed (solid line). HE stain 10x.

Figure D. Grade 2: Moderate macrophage infiltrate foci (circle) in the lamina propria with MGC inside, and separation of the Lieberkühn crypts (arrows). HE stain 10x.
Figure E. Grade 3: Ileum sample with multiple foci of macrophage infiltrate (circles) in the lamina propria of the intestinal mucosa. Some of these foci infiltrate the mucosa between the crypts of Lieberkühn, causing a gap between them. Additionally, MGC (arrows) are present in the mucosa. HE stain 10x.
Figure F. Grade 3: Ileum sample of macrophage infiltrate in the lamina propria with multiple MGC (arrow). Ziehl–Neelsen (ZN) stain 10x
Figure G. Grade 4, Ileum sample with diffuse macrophage infiltrate (circles) in the lamina propria of the intestinal mucosa. In addition to a marked atrophy and fusion of intestinal villi. HE stain 4x.
Figure H (zoom of Figure G): Grade 4, Ileum sample with diffuse macrophage infiltrate (circles) in the lamina propria of the intestinal mucosa with fusion of the intestinal villi. HE stain 10x.
Figure I. Grade 5: Ileum sample with diffuse macrophage infiltrate in the lamina propria, presenting gaps between Lieberkühn crypts and atrophy and fusion of intestinal villi. In addition, an organized granuloma is observed. ZN stain 4x.
Figure J. Grade 5: Ileum sample presenting marked atrophy and fusion of intestinal villi. HE 4x.
