# Supplementary material for: Association between the severity of histopathological lesions and Mycobacterium avium subspecies paratuberculosis (MAP) molecular diversity in cattle in southern Chile
Source: Front Vet Sci. 2023 Jan 12;9:962241. doi: 10.3389/fvets.2022.962241 (PMC9878319; doi:10.3389/fvets.2022.962241)
Supplement: Supplementary file 2 [file Data_Sheet_2.DOCX]

Supplementary Material B

Histopathological severity score (HSS) distribution (median, minimum (Min), and maximum (Max). Average optical density (OD) for each haplotype

| **Haplotype** | **Frequency** | **Ileum** | | **MLN** | | **OD** |
| --- | --- | --- | --- | --- | --- | --- |
|  |  | **Median** | **Min - Max** | **Median** | **Min - Max** |  |
| A | 39 | 1 | 0 - 5 | 1 | 0 - 5 | 148.8 |
| B | 10 | 1 | 0 - 5 | 1 | 0 - 5 | 163.1 |
| C | 2 | 2.5 | 1 - 4 | 3 | 1 - 5 | 124.1 |
| D | 2 | 0.5 | 0 - 1 | 0.5 | 0 - 1 | 85.5 |
| E | 3 | 1 | 0 - 2 | 0 | 0 - 1 | 106.5 |
| F | 2 | 0 | 0 | 0.5 | 0 - 1 | 67.5 |
| G | 2 | 2 | 0 - 4 | 2 | 0 - 4 | 85.5 |
| H | 1 | 0 | - | 0 | - | 52 |
| I | 1 | 0 | - | 1 | - | 228 |
| J | 4 | 0 | 0 - 3 | 0 | 0 - 1 | 100.5 |
| K | 2 | 1.5 | 0 - 3 | 0.5 | 0 - 1 | 45 |
| L | 2 | 0.5 | 0 - 1 | 0.5 | 0 - 1 | 65.5 |
| M | 1 | 2 | - | 2 | - | 102 |
| N | 1 | 1 | - | 1 | - | 157 |
| O | 1 | 0 | - | 0 | - | 128 |
| P | 2 | 0.5 | 0 - 1 | 1 | 1 - 1 | 172.5 |
| Q | 1 | 5 | - | 1 | - | 189 |
| R | 1 | 3 | - | 0 | - | 183 |
| S | 2 | 3 | 1 - 5 | 2 | 1 - 3 | 212 |
